# Lipid Peroxidation in Neurodegeneration

**DOI:** 10.3390/antiox10030484

**Published:** 2021-03-19

**Authors:** Consuelo Cháfer-Pericás

**Affiliations:** Health Research Institute La Fe, 46026 Valencia, Spain; m.consuelo.chafer@uv.es

Neurodegenerative diseases have multiple social and economic impacts on society, and they are the cause of millions of deaths every year. The potential molecular mechanisms that generate these pathologies have been widely studied. Impaired lipid metabolism, particularly lipid peroxidation, plays an important role in the development of many neurodegenerative diseases, including Alzheimer’s disease, Parkinson’s disease, and amyotrophic lateral sclerosis. In general, these diseases showed impaired levels of lipid peroxidation products in comparison with healthy elderly participants.

The aim of this Special Issue “Lipid Peroxidation in Neurodegeneration” is to advance the knowledge of the physiopathological mechanisms involved in the development of complex neurodegenerative diseases. Specifically, lipid peroxidation plays an important role, as brain has a rich composition of lipids. Additionally, the relationship between lipid peroxidation and some antioxidants’ action has been largely evaluated. However, some controversial results were obtained, and further studies are required. In addition, this Special Issue is focused on potential early lipid peroxidation biomarkers to detect neurodegenerative diseases.

In this sense, the inhibition of neuronal oxidation could avoid disease progression, as well as reducing its severity. A review of promising antioxidant agents (polyphenols, antioxidant vitamins…) was carried out, showing some potential benefits of antioxidant supplementation [1].

Another review focused on the capacity of neuromelanin as an antioxidant, and its involvement in oxidative stress, depending on the neuromelanin saturation state and its extracellular release [2].

Specifically, the mechanisms that could relate lipid peroxidation with neurodegeneration are ferroptosis [3] and mitochondrial dysfunction. According to this, a research study was carried out evaluating changes to oxidative stress and mitochondrial DNA copy numbers in patients with Parkinson’s disease under dopamine therapy [4]. This study measured mitochondrial copy numbers and thiobarbituric acid reactive substances (TBARS).

In addition, lipid peroxidation compounds, such as isoprostanes, neuroprostanes, prostaglandines, and dihomo-isoprostanes, could be promising early neurodegenerative diseases biomarkers. In fact, previous studies showed a relationship between lipid peroxidation compounds and Alzheimer’s disease. However, correlations between plasma and cerebrospinal fluid samples were not satisfactory for any of the studied lipid peroxidation compounds [5].

Previous studies showed the utility of lipid peroxidation compounds in minimally invasive samples, such as plasma. Actually, they could be good indicators of the effectiveness of different treatments and they could serve as diagnostic and/or prognostic biomarkers. In this sense, lipid peroxidation needs to be evaluated in relation to different neurodegenerative conditions, as well as from different points of view.

Regarding Alzheimer’s disease, its differential diagnosis is a complex task due to its clinical similarity to other neurodegenerative diseases. Therefore, the clinical validation of potential Alzheimer’s disease-specific biomarkers in minimally invasive samples constitutes a great challenge in early diagnosis [6].

To conclude, more studies about the relationship between lipid peroxidation and neurodegenerative diseases are required, since they could provide promising results in the development of new therapeutic targets.
